# The past, present, and future of ecogeographic isolation between closely related *Aquilegia* plants

**DOI:** 10.1002/ece3.10098

**Published:** 2023-05-25

**Authors:** Yulin Weng, Huiqiong Li, Jiqin Yang, Zhi‐Qiang Zhang

**Affiliations:** ^1^ Yunnan Key Laboratory of Plant Reproductive Adaptation and Evolutionary Ecology, School of Ecology and Environmental Sciences, Institute of Biodiversity Yunnan University Kunming China; ^2^ College of Environment and Ecology Xiamen University Xiamen China; ^3^ Gansu Liancheng National Nature Reserve Lanzhou China

**Keywords:** climate change, ecological niche models, reproductive isolation, speciation

## Abstract

Quantifying the strength of the ecogeographic barrier is an important aspect of plant speciation research, and serves as a practical step to understanding the evolutionary trajectory of plants under climate change. Here, we quantified the extent of ecogeographic isolation in four closely related *Aquilegia* species that radiated in the Mountains of SW China and adjacent regions, often lacking intrinsic barriers. We used environmental niche models to predict past, present, and future species potential distributions and compared them to determine the degree of overlap and ecogeographic isolation. Our investigation found significant ecological differentiation in all studied species pairs except *A. kansuensis* and *A. ecalacarata*. The current strengths of ecogeographic isolation are above 0.5 in most cases. Compared with current climates, most species had an expanding range in the Last Glacial Maximum, the Mid Holocene, and under four future climate scenarios. Our results suggested that ecogeographic isolation contributes to the diversification and maintenance of *Aquilegia* species in the Mountains of northern and SW China and would act as an essential reproductive barrier in the future.

## Graphical abstract

Ecogeographic isolation is an important reproductive barrier, but was rarely included as a component of reproductive isolation in previous studies. We found that ecogeographic isolation played a vital role in speciation and maintaining species boundaries in *Aquilegia* species.

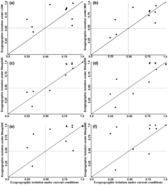

## 1 INTRODUCTION

Understanding the relative contribution of barriers in restricting gene flow between taxon has been a major goal of recent speciation research in plants. Reproductive barriers in plants typically act sequentially throughout the life cycle of organisms in nature to reduce gene flow between lineages (Baack et al., 2015; Christie et al., 2022; Lowry et al., 2008). The earliest‐acting barrier is ecogeographic isolation, i.e., taxa occupy different geographic ranges result of adapting to different environments, disproportionately impacting total reproductive isolation (Kay, 2006; Ramsey et al., 2003; Schemske, 2010; Sobel & Chen, 2014). Hence, quantifying ecogeographic isolation is essential to understand evolutionary forces during species divergence in plants.

Ecogeographic barriers can promote speciation has a long history (Lowry, 2012; Stebbins, 1950). However, it has rarely been documented in previous studies that quantifying multiple reproductive barriers in plants (Lowry et al., 2008), largely owing to methodological challenges to complete sampling when measuring ecogeographic isolation. Nevertheless, it can be partially resolved through models of species' ranges using soil and climate data (e.g., Li et al., 2018; Sobel & Chen, 2014), and then compared with each other to determine the degree of ecogeographic isolation (Reviewed in Baack et al., 2015). For example, Sobel and Chen (2014) evaluated the ecogeographic isolation from 12 pairs of monkeyflowers (*Mimulus*) using ecological niche models and found this approach can provide a reliable measure of ecogeographic isolation. Moreover, ecological niche models (ENMs) have been widely used in estimating species potential distributions under past and future climates from contemporary distributions (Cozzolino et al., 2021; Sobel & Chen, 2014; Tian et al., 2020; Wan et al., 2021), suggesting we can capture past and future potential ranges of species using the available abiotic variable data. Furthermore, since species are shifting their distributions in response to ongoing climate change (Chen et al., 2011; Pecl et al., 2017), geographically isolated closely related plants might shift or expand their ranges and start to overlap. By modeling species distribution, Duffy and Jacquemyn (2019) foretasted shifts in geographic distributions in response to climate change and predicted how climate change would impact ecogeographic isolation between related *Pulmonaria* species, highlighting that climate change can potentially affect the evolutionary trajectory of plants in the future. Likewise, it is also interesting to predict the past distribution of different taxa using ecological niche models and explore the historical importance of ecogeographic isolation during the early stage of speciation.

**FIGURE 1:**
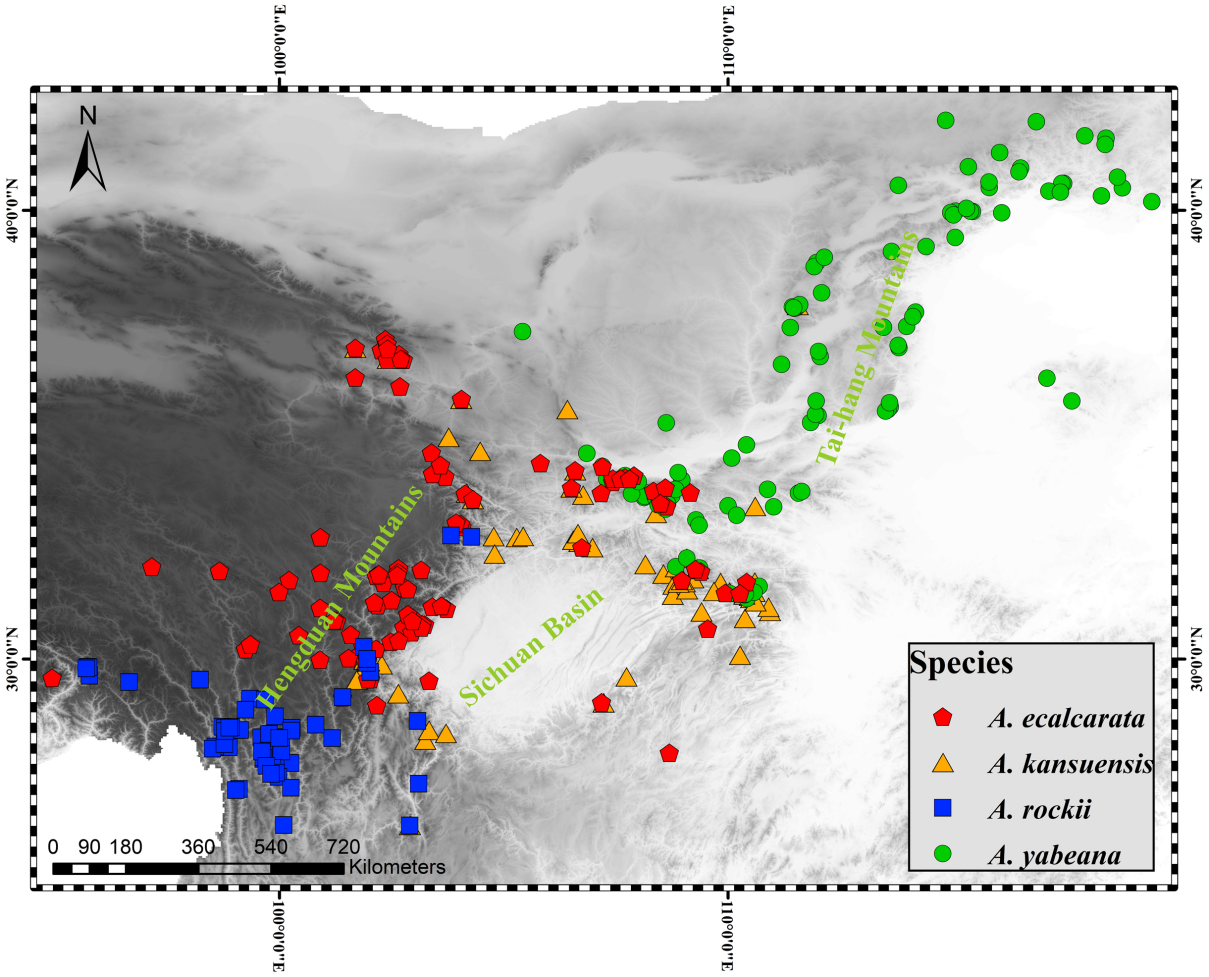
The distribution of each species of *Aquilegia ecalcarata*, *A. kansuensis*, *A. rockii*, and *A. yabeana* in China, among which the red dots represent *A. ecalcarata*. The darkest shading on the map indicates area with the highest elevation.

The columbines genus *Aquilegia* L. contains about 70 species widely distributed across temperate regions of Eurasia and North America (Munz, 1946). As a classic example of adaptive radiation (diversification from an ancestral species that produces descendants adapted to use a great variety of distinct ecological niches), *Aquilegia* has offered great opportunities to learn about the process of speciation (Hodges et al., 2003; Kramer, 2009). From a common ancestor in Asia, one radiation occurred in North America via Northeastern Asian precursors, and another radiation took place in central and western Asia and Europe (Bastida et al., 2010; Fior et al., 2013). Adaptation to different habitats was thought to be the main force driving both radiations (Bastida et al., 2010; Li et al., 2014). Therefore, we speculated that ecogeographic isolation was strong between *Aquilegia* species. Although many studies on pollinator isolation of *Aquilegia* species (Grant, 1952, 1976, 1992; Hodges & Arnold, 1994; Tang et al., 2007), none of them explored the potential role of ecogeographic isolation in *Aquilegia* species. *Aquilegia yabeana* Kitag, *A. rockii* Munz, *A. kansuensis* Erst (*Aquilegia oxysepala* var. *Kansuensis* Brühl) and *A. ecalcarata* Maxim, distributed in northern and southwestern China, comprise a monophyletic clade: *A. yabeana* is sister to the rest of the species and *A. ecalcarata* is the latest derived one (Fior et al., 2013). The different distribution pattern (Figure 1) brings about interesting questions: does the early‐acting ecogeographic isolation contribute to diversification in these *Aquilegia* species? Therefore, we hypothesized that ecogeographic isolation, species adapted to different habitats, played an essential role in the origin of reproductive isolation, and will reduce gene flow among related columbines in the future. To test the hypothesis, we investigate ecogeographic isolation between four very closely related *Aquilegia* species in China using ENMs under past, present conditions, and four plausible climate change scenarios.

## 2 MATERIALS AND METHODS

### 2.1 Species and occurrence data

All studied columbines are perennial herbs endemic to mountains of northern and SW China, primarily distributed in the Hengduan Mountains, Tai‐hang Mountains, and mountains of Sichuan Basin (Figure 1). These four species distribute differently: *A. yabeana* occurs mainly in the mountains between 1000 and 2000 m a.s.l. in northern China; *A. kansuensis* mainly distributes in the mountains between 2500 and 3500 m a.s.l. in northwestern China; *A. rockii* is primarily at 2500–3500 m a.s.l. in mountains of Southwestern China; *A. ecalcarata* is largely overlapped with *A. kansuensis* while sharing a very limited range with *A. yabeana* and *A. rockii* (Huang et al., 2018; Figure 1). In the species' ranges of *A. yabeana*, *A. kansuensis*, *A. rockii*, and *A. ecalcarata*, the annual mean precipitation (mm) are 674.83, 985.89, 744.13, and 755.96, respectively. The annual mean temperature (°C) of them are 7.36, 8.54, 4.12, and 4.77, respectively (our unpublished data).

We collected species occurrence from field investigations, references, the Chinese Virtual Herbarium (CVH), and the Global Biodiversity Information Facility (GBIF). The occurrence records from the field investigation were collected from 2018 to 2020 under the joint efforts of our research team members. We used “*Aquilegia yabeana*,” “*Aquilegia rockii*,” “*Aquilegia oxysepala* var. *kansuensis*,” and “*Aquilegia ecalcarata*” as keywords to search plant collection records on GBIF (https://www.gbif.org; *A. ecalcarata*, *A. oxysepala* var. *kansuensis*, *A. yabeana*, and *A. rockii*: https://doi.org/10.15468/dl.wqtv6e, 29 Nov 2022) and CVH (http://www.cvh.ac.cn/). Incomplete, duplicated, and ambiguous identified occurrence records were excluded. We then plotted the remaining records on a global map with a resolution of 2.5 arc‐min using ArcGIS 10.5 (Environmental Systems Research Institute), excluding points that were irrational (i.e., on rivers, oceans, or outside the known range). In cells with multiple records, only one point was retained. Finally, we included 335 occurrence records (73 of *A. kansuensis*, 105 of *A. ecalcarata*, 56 of *A. rockii*, and 101 of *A. yabeana*) for subsequent analyses. Our study region was restricted to the range of the Hengduan Mountains, Tai‐hang Mountains, and the Sichuan Basin (91°–125° E and 20°–47° N) to ensure georeferenced specimen sampling and consistent abiotic layers used for ENMs.

### 2.2 Environmental variables

We downloaded 19 bioclimatic variables of current environmental variables from WorldClim (a database of high spatial resolution global weather and climate data, http://world clim.org/) with 2.5 arc‐min resolution (Hijmans et al., 2005). Monthly climate data for minimum, mean, and maximum temperature, precipitation, solar radiation, wind speed, water vapor pressure, and total precipitation were also downloaded from WorldClim (Fick & Hijmans, 2017). To avoid collinearity between variables, we selected environmental variables with Pearson's correlation coefficient *R* < .75 and Variance Inflation Factor (VIF) > 10 (Dormann et al., 2013) across all variables prior to modeling. Consequently, we retained six environmental variables to construct the species distributions, including solar radiation of December (srad12) and five bioclimatic variables (annual mean temperature [bio1], isothermality [bio3], temperature annual range [bio7], annual precipitation [bio12], and precipitation seasonality [bio15]).

The potential distribution of each species in the future and the past were projected based on the above five bioclimatic variables in each climate change scenario downloaded from WorldClim (Hijmans et al., 2005). We selected two SSP (Shared Socioeconomic Pathways) scenarios (ssp245 and ssp585) for years 2050 (average of 2041–2060) and 2090 (average of 2081–2100) and two historical periods of the Last Glacial Maximum (LGM; approximately 22,000 years ago) and the Mid Holocene (MID; approximately 6000 years ago) to predict the future and past distributions of each species, respectively. For each SSP scenario, three general circulation models (GCMs) were considered for projection and assembling, including CCSM4, MIROC, and MPIESM‐P. For highly variable when using different GCMs (Figure S1), we retained and assembled two GCMs of CCSM4 and MPI‐ESM‐P for a high rate of area overlap (Figure S2). The values of mean annual mean temperature and mean annual mean precipitation were extracted from LGM to four future climate change scenarios.

### 2.3 Habitat divergence and niche identity

To test the current habitat differentiation of *A. yabeana*, *A. rockii*, *A. kansuensis* and *A. ecalcarata*, we analyze habitat differentiation without a priori designation of the regions using principal component analysis (PCA) in the R package “ggfortify” (Horikoshi & Tang, 2016) based on the above six environmental variables. In addition, the discriminant function analysis (DFA) in the R package ‘MASS’ (Ripley et al., 2013) was also applied to further test habitat differentiation.

To quantify the degree of habitat differentiation and environmental space identity, we used Schoener's *D* (Schoener, 1968) and Warren's *I* (Warren et al., 2008) implemented in R package ‘ENMTools’ (Warren et al., 2010). The two indexes range from 0 (complete divergence or no overlap) and 1 (high similarity or complete overlap). The observed value of Schoener's *D* and Warren's *I* were estimated by layers generated based on the GLM model and the expected value of each index simulated from 100 pseudoreplicates. A nonparametric Monte Carlo test was used to examine the difference between the observed and the expected index. The differences were considered to be statistically significant when *p* < .05. The niche breadth of each studied species was calculated in the R package “ENMTools” (Warren et al., 2010) to test niche breadth for each species with Levins'*B* (Levins, 1968). The value of Levins'*B* was generated using layers based on the GLM model by bootstrap sampling. A Mann–Whitney *U* test in R was used to test whether niche breadth is significantly different.

### 2.4 ENMs and ecogeographic isolation

We used the R package ‘Biomod2’ to select the best model for ENMs (Thuiller et al., 2019). To improve the accuracy of ENMs, 200,000 pseudo‐absence coordinates were randomly outputted using the R package “dismo” (Hijmans et al., 2017) based on the distribution range of *Aquilegia*. To assess model performance, all occurrence sites of each species were randomly partitioned into training data set (70%) and testing data set (30%) following Li et al. (2020). The training data set was used to model calibration, and the testing data set was applied to cross‐validation of the model evaluation during the calibration process. Each model in the R package “Biomod2” ran 100 replicates with a resampling method to subsampling. ROC (Receiver Operating Characteristic [ROC] and True Skill Statistic [TSS]) were used to measure the accuracy of models (Allouche et al., 2006; Fawcett, 2006). A perfect model would with both ROC and TSS values of 1. It is generally recognized that an excellent model performance with ROC > 0.9 and TSS > 0.8. Finally, GLM, MARS, and Maxent had high values of ROC and TSS in all models and were selected as optimal models for ENMs (Figure S3).

To construct the distribution range of each species at current conditions, three optimal models and 100 replicates of each model were performed for each species. A total of 300 layers for each species were assembled with a threshold of TSS ≥ 0.8 according to proportion in R package “Biomod2.” Ensembled layers were constructed and then mapped by ArcGIS 10.5. The value of the cutoff that was generated when the finish model running was used as the threshold to determine the extent of suitable habitat, the region that probability of a species below cut‐off would be believed to be unsuitable habitat and subtracted using ArcGIS 10.5. Finally, areas of the distribution for each species at current conditions were calculated. For predicting the distribution of each species in all climate change scenarios and past climate scenarios, we trained the model using current bioclimatic variables and projected it with future and historical bioclimatic variables.

Ecogeographic isolations at past climate scenarios, current, and all climate change scenarios were calculated pairwise using the equation:

$${\mathrm{RI}}_{\text{Ecogeo}}=1-S/\left(S+U\right).$$

where *S* refers to the shared number of cells in which both species are predicted to occur and *U* refers to the unshared areas of suitable habitat for a given species (Sobel & Chen, 2014). Ecogeographic isolation values range from 0 (the entire extent of habitat shared) to 1 (completely separated). RI_Ecogeo_ can only be interpreted as a pairwise measure because ecogeographic isolation is asymmetric and unique in the amount of suitable habitat between two species. Therefore, ecogeographic isolation in this monophyletic clade was calculated in pairs for past, present, and all climate change scenarios in the future, separately.

## 3 RESULTS

### 3.1 Habitat divergence, niche identity, and ecogeographic isolation in the present

**FIGURE 2:**
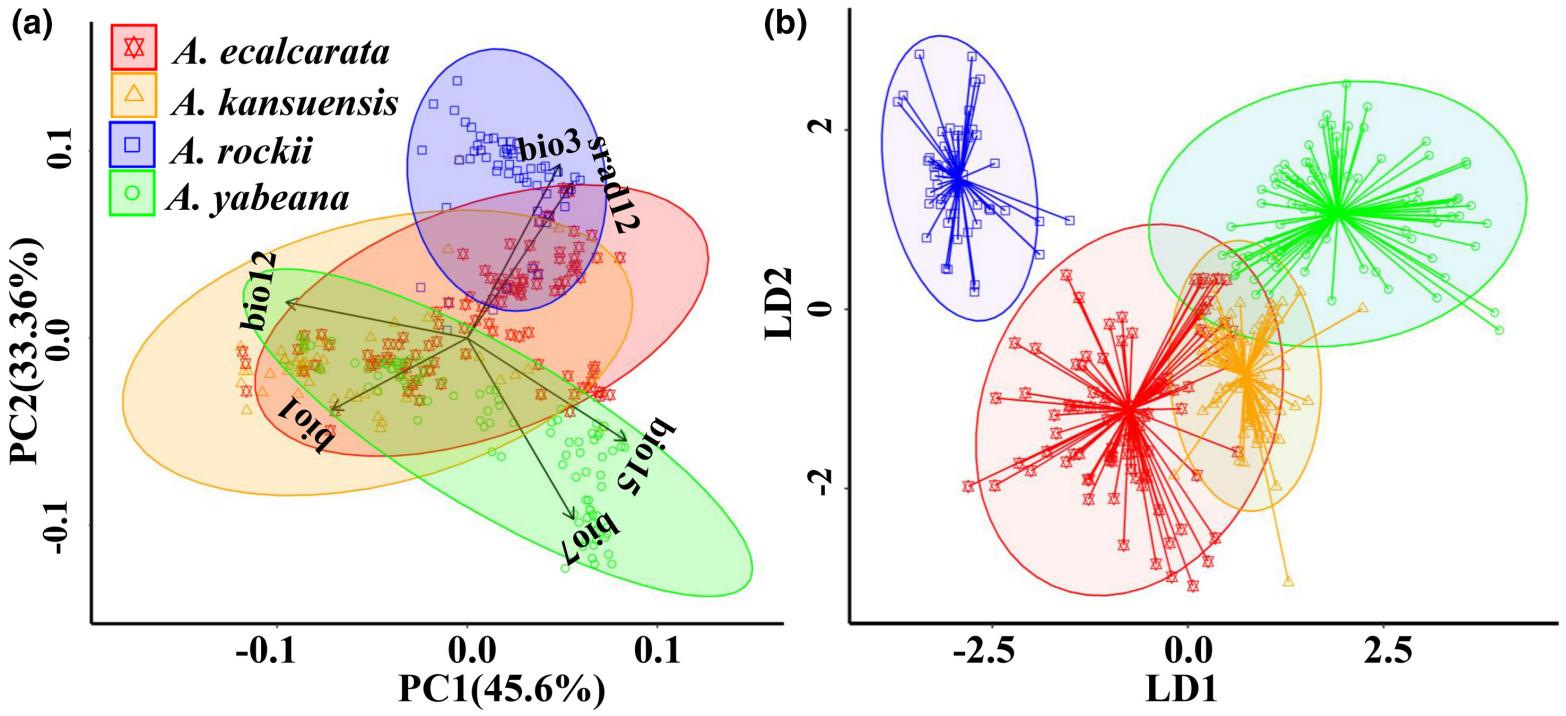
Principal component analysis (a) and discriminant function analysis (b) of environmental factors of *Aquilegia ecalcarata*, *A. kansuensis*, *A. rockii*, and *A. yabeana*.

In PCA of the six environmental variables, the first two components explained 45.06% and 33.36% of the variation for the 335 occurrence points, respectively (Figure 2a). PC1 was positively correlated with precipitation seasonality (bio15) and temperature annual range (bio7), negatively correlated with annual precipitation (bio12) and annual mean temperature (bio1), while PC2 was mainly correlated with isothermality (bio3) and solar radiation of December (srad12; positively) and temperature annual range (bio7) (negatively; Table S2). The result of PCA showed *A. yabeana* and *A. rockii* were entirely separated in the biplots, but these *Aquilegia* species mixed with each other at different levels in other cases. Among them, *A. ecalcarata* and *A. kansuensis* overlapped at most points. Similarly, the result of the DFA showed *A. ecalcarata* and *A. kansuensis* largely mixed, while others were separated with varying degrees of overlaps.

**FIGURE 3:**
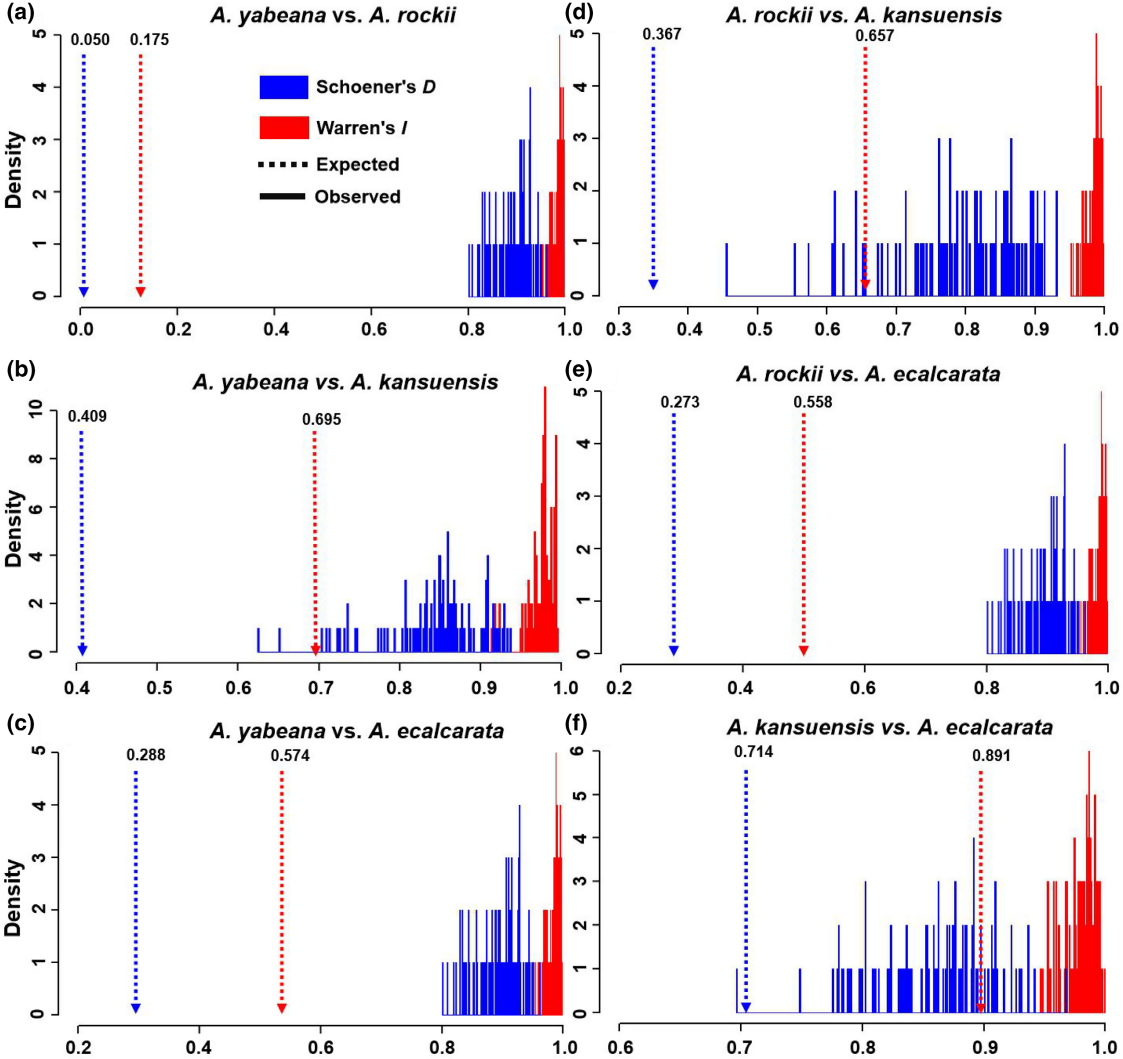
Niche identity test of *Aquilegia ecalcarata*, *A. kansuensis*, *A. rockii*, and *A. yabeana*. The vertical dotted line showing the empirical value of Warren's *I* and Schoener's *D* and histograms represent the frequency of expected. Warren's *I* and Schoener's *D*. The null hypothesis of identical niche is rejected if the vertical dotted line falls outside the 95% probability threshold of the expected distributions (*p* < .05). (a) Niche identity test for *A. yabeana* and *A. rockii*; (b) Niche identity test for *A. yabeana* and *A. kansuensis*; (c) Niche identity test for *A. yabeana* and *A. ecalcarata*; (d) Niche identity test for *A. rockii* and *A. kansuensis*; (e) Niche identity test for *A. rockii* and *A. ecalcarata*; (f) Niche identity test for *A. kansuensis* and *A. ecalcarata*.

Consistent with the results of PCA and DFA analysis, the niche identity test showed five of six species pairs rejected the hypothesis that environmental niches between species pairs were identical (*p* < .05, Figure 3a–e) based on the niche similarity index (Schoener's *D* and Warren's *I*). Only the species pair of *A. ecalcarata* and *A. kansuensis* could not reject the hypothesis that environmental niches were identical (Figure 3f). Based on Levins'*B* values, the niche breadth was significantly different among species except species pair *A. ecalcarata* and *A. kansuensis*, consistent with results of PCA, DFA, and niche identity test, supporting that habitat differentiation in most species pairs except species pair *A. ecalcarata* and *A. kansuensis*.

The current distribution models showed the degree of overlap between six species pairs ranged from complete allopatry in *A. rockii* and *A. yabeana* to intermediacy in most cases and nearly complete sympatric, as in *A. ecalcarata*, *A. kansuensis* (Figure S6). Correspondingly, RI_ecogeography_, an index of the degree of ecogeographic isolation, was 1 in *A. rockii* and *A. yabeana* (Table 1). On the contrary, RI_ecogeography_ was 0.3249 and 0.3399 in *A. ecalcarata* and *A. kansuensis*, respectively (Table 1). In addition, the habitat of *A. yabeana* nearly no overlap with the other two species (Figure S6b,c). *A. rockii* also had a relatively independent habitat (Figure S6a,d,e) but partially overlapped with *A. ecalcarata* (Figure S6e, RI_ecogeo; *A. rockii*_ = 0.2565, RI_ecogeo; *A. ecalcarata*_ = 0.7748; Table 1).

**TABLE 1:** Ecogeographical isolation values between species pairs of *Aquilegia ecalcarata*, *A. kansuensis*, *A. rockii*, and *A. yabeana* under current environmental conditions.

Ecogeographical isolation				Species pairs
*A. ecalcarata*	*A. kansuensis*	*A. rockii*	*A. Yabeana*	
—	0.3399	0.2565	0.7865	*A. ecalcarata*
0.3249	—	0.5627	0.7817	*A. kansuensis*
0.7748	0.8705	—	1.0000	*A. rockii*
0.8595	0.8595	1.0000	—	*A. yabeana*

### 3.2 Ecogeographic isolation under all climate scenarios

**FIGURE 4:**
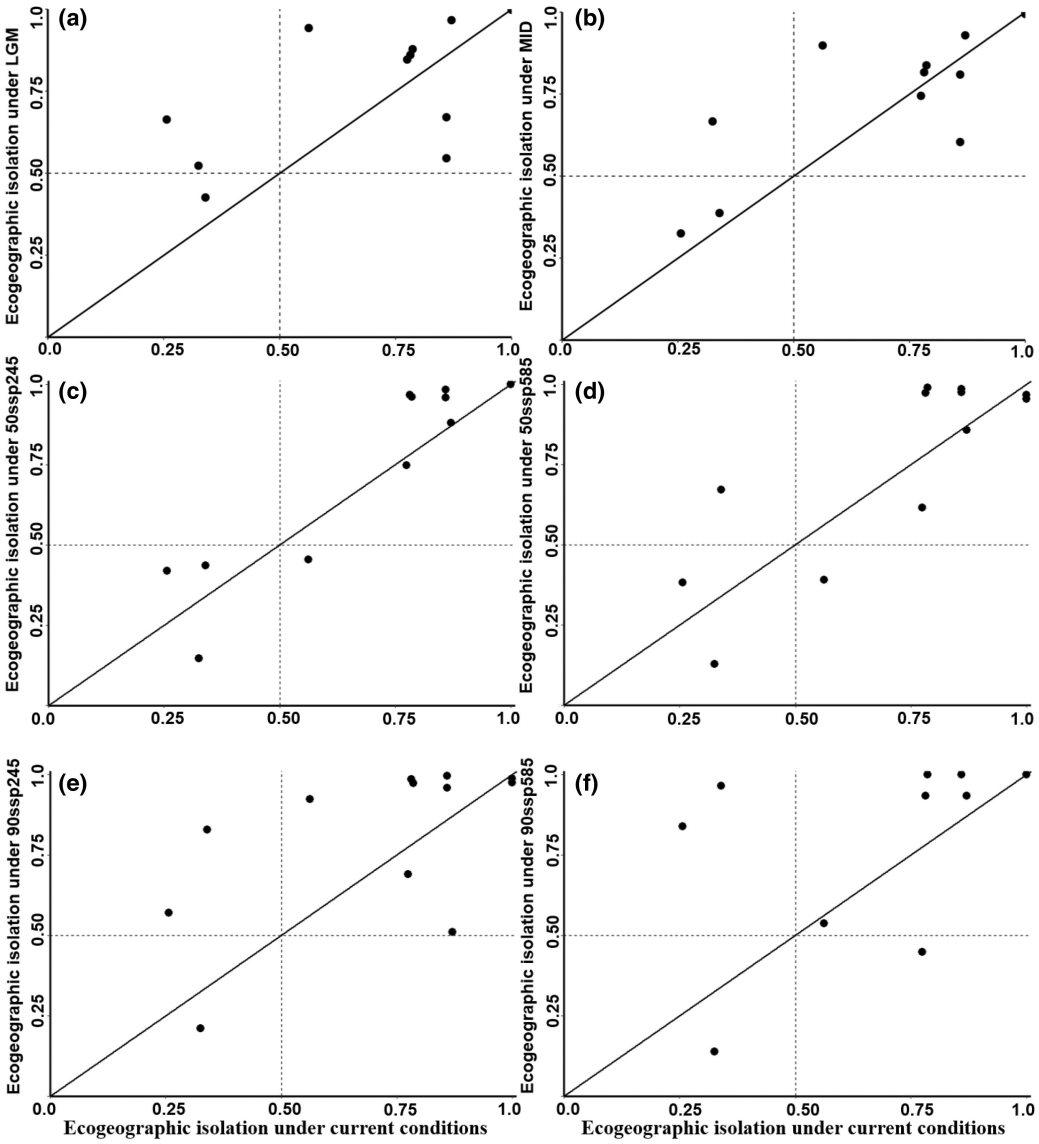
Pairwise values of ecogeographical isolation of *Aquilegia ecalcarata*, *A. kansuensis*, *A. rockii*, and *A. yabeana* at current with past of Last Glacial maximum (LGM) and Mid Holocene (MID), different scenarios in future: (a) LGM, (b) MID, (c) the 50ssp245, (d) 50ssp585, (e) 90ssp245, (f) 90ssp585. The line indicates no change in the ecogeographic isolation of species pairs. Values below the line indicate decreased ecogeographic isolation, while values above the dashed line indicate increased ecogeographic isolation.

Under past climate scenarios, all studied Aquilegia species, with the exception of *A. rockiia*, exhibited an expansion in predicted ranges. In contrast, *A. rockii*a experienced a slight contraction in the predicted habitats area in the MID (Table 2). All four species in the LGM had expanded habitats compared with the MID. Of them, with *A. yabeana* occuping the largest habitats areas (13.13 × 10^5^ km^2^). *Aquiliegia ecalcarata* and *A. kansuensis* had similar habitats areas under past scenarios in the same periods. Meanwhile, *A. rockii* maintained stable habitat areas (Table 2). Compared with the current condition, the average strengths of ecogeographic isolation between species pairs increased with range expansion in the LGM and MID, with species with low RI_ecogeo_ under current conditions increasing the most under past climate scenarios (Figure 4a,b).

**TABLE 2:** Dynamics of the predicted habitats areas of *Aquilegia ecalcarata*, *A. kansuensis*, *A. rockii*, and *A. yabeana* under past scenarios and all climate change scenarios compared with current.

Species	LGM	MID	Current	50ssp245	50ssp585	90ssp245	90ssp585
*A. ecalcarata*	9.05 [+2.33]	8.4 [+1.68]	6.72	4.57 [−2.15]	3.88 [−2.84]	3.24 [−3.48]	0.8 [−5.92]
*A. kansuensis*	9.14 [+2.27]	7.68 [+0.81]	6.87	8.94 [+2.07]	10.28 [+3.41]	14.83 [+7.96]	18.77 [+9.9]
*A. rockii*	2.45 [+0.42]	1.9 [−0.13]	2.03	1.98 [−0.05]	2.41 [+0.38]	2.33 [+0.3]	2.72 [+0.69]
*A. yabeana*	13.13 [+8.71]	6.47 [+2.05]	4.42	4.74 [+0.32]	5.35 [+0.93]	4.87 [+0.45]	2.65 [−1.77]

*Note*: The unit is 10^5^ km^2^, (+) means area of habitats gain compared with Current, (−) means area of habitats loss.

Abbreviations: 50ssp245, Shared Socio‐economic Pathways 245 in 2050; 50ssp585, Shared Socio‐economic Pathways 585 in 2050; 90ssp245, Shared Socio‐economic Pathways 245 in 2090; 90ssp585, Shared Socio‐economic Pathways 585 in 2090; LGM, Last Glacial Maximum; MID, Mid Holocene.

Under the different future climate change scenarios, the predicted habitat areas of species also changed a lot. For example, the potential habitat areas of *A. ecalcarata* decreased from 6.72 × 10^5^ km^2^ at current to 0.8 × 10^5^ km^2^ under scenarios of Shared Socio‐economic Pathways 585 in 2090. However, the potential habitat areas of *A. kansuensis* increased to 18.77 × 10^5^ km^2^ in the same scenario. *Aquilegia yabeana* maintained relatively stable habitat areas in the future, which differed from its performance in LGM or MID. *Aquilegia rockii* had stable habitat areas all the time. Consequently, significant changes in ecogeographic isolation were predicted to occur in the future. Compared with current climates, the average strengths of ecogeographic isolation between different species pairs were predicted to increase under future scenarios investigated, but there were no apparent increases under ssp585 in 2050 (Figure 4c–f). Similar to past scenarios, species with low RI_ecogeo_ under current conditions generally increased the most under all future climate change scenarios.

## 4 DISCUSSION

### 4.1 Present species distribution and ecogeographic isolation

Ecogeographic isolation refers to the degree to which a species is geographically isolated from its close relatives due to its habitat preference (Sobel et al., 2010). For example, summer drought in the inland habitat and salty soil in the coastal habitat act as barriers to the immigration of *Mimulus guttatus* between the coast and inland populations (Lowry et al., 2008). Our findings suggested that a complex set of factors, including temperature, precipitation, and solar radiation, shapes the distribution differences among studied *Aquilegia* species (Figure 2; Table S2). Specifically, temperature and precipitation were mainly correlated with both PC1 and PC2, suggesting they play a vital role in shaping the distribution difference among these *Aquilegia* species. The result was consistent with other studies that found precipitation, temperature, and solar radiation are the primary environmental factors affecting plant distribution (Bennie et al., 2008; Zhong et al., 2010). The importance of precipitation and temperature in determining plant range limits under climate change has also been established by recent research (Dixon & Busch, 2017). Moreover, the results of PCA and DFA showed that most studied *Aquilegia* species pairs occupied significantly different niches, except for one species pair (*A. kansuensis* and *A. ecalacarata*; Figure 3; Figure S6). This finding further supports the role of ecogeographic isolation as a reproductive barrier between most species pairs. It is consistent with a previous study on 16 species pairs of western North American monkey flowers that found niche divergence decreased with increasing range overlap (Sobel & Chen, 2014).

Theoretically, when ecogeographic isolation surpasses 0.5, later‐acting barriers cannot exceed its relative impact on total gene flow (Ramsey et al., 2003). Given the existence of fine‐grained environmental variation, we hypothesized that habitat adaptation could explain the variance in the distribution of closely related *Aquilegia* species. Our analysis revealed that ecogeographic isolation was crucial among 9 out of 12 possible species pairs, as all their isolation strengths were above 0.5 (Table 1; Figure S6). This echoes past evidence that adaptation to local environments contributes to higher ecogeographic isolation (e.g., see Duffy & Jacquemyn, 2019; Glennon et al., 2012; Lowry et al., 2008; Sobel & Chen, 2014). However, closely related sister species *A. kansuensis* and *A. ecalacarata* have largely overlapping distributions, resulting in moderate ecogeographic isolation between them (Table 1). In our field investigations, we also found that these two species are often sympatrically distributed in natural populations. Our findings contrast with Jordan's rule, which predicted that the most closely related species are spatially proximate (Jordan, 1908; Sobel & Chen, 2014). It can partly be explained by the fact that current distributions between related species pairs owe not only to contemporary ecological factors, but also to their evolutionary history. Young species may converge in habitat usage due to their co‐evolutionary history. For example, sympatry among sister taxa has higher range overlaps than non‐sister congener comparisons (Anacker & Strauss, 2014; Grossenbacher et al., 2014). Alternatively, the dispersal capacity of a given species and interactions with predators, competitors, and mutualists may also strongly affect its ecological niche, species range, and the resulting ecogeographic isolation (Barvea et al., 2011; Dool et al., 2022).

### 4.2 Ecogeographic isolation in the past

The investigation of global climate change and the response of species distribution provides a unique window into the evolutionary history and the evolutionary trajectory in the future. Paleo‐climatic records offer glimpses into the past climate, giving insight into what species underwent. For example, average temperatures in the LGM were substantially colder than they are today, and became increasingly hot after the LGM, reaching a temperature peak near the MID (Otto‐Bliesner et al., 2006; Renssen et al., 2009; Wanner et al., 2008). The dramatic fluctuations in climate had a significant impact on species distributions, resulting in fragmented habitats among populations or more overlapping in species ranges between relatives (Hewitt, 1999; Stone & Wolfe, 2021). So we expected that the strength of the ecogeographic barrier between related species could be dynamic and change over time as a consequence of climate changes.

The general view is that species in temperate climates tend to contract their ranges in refugial areas during glacial expansion and expand following climate warming. Unlike the classical paradigm of temperate species' responses to glaciation, we found that only one species, *A. rockii*, contracted its ranges to small pockets of suitable habitat, while the other three species experienced an increase in suitable habitat during the LGM relative to the present day. A recent study on *Penstemon* species in the Pacific Northwest of North America also experienced increased suitable habitat during the GLM, resulting in gene exchange between species (Stone & Wolfe, 2021). However, our study found that four studied *Aquilegia* species respond differently to GLM, and the area of overlap in distributions did not increase largely or decrease as species expanded southern or lower latitude. For example, *A. kansuensis* and *A. ecalcarata* decreased overlap in GLM relative to the present day (Figure S5). This resulted in the degrees of ecogeographic isolation in GLM is larger than that under current climates in most cases (Figure 4). Furthermore, a recent study on *Dipelta* sister species in the same area also revealed that two adjacently distributed species became divergent in distributions following expansion during the LIG–LGM period (Tian et al., 2019). After the LGM, suitable habitat for all species shifted northward and restricted considerably, with the degree of ecogeographic isolation between most species pairs during the Mid‐Holocene warm period appearing similar to their strength in GLM (Figures S7–S9).

Our study revealed that the ecogeographic isolation between most species pairs experienced a strength greater than 0.5 in both GLM and MID (Figure 4). Based on our genome sequencing data, we found that four studied *Aquilegia* species form a distinct monophyletic clade at ~2.68 Ma and other species split from *A. yabeana* at ~1.74 Ma (our unpublished data), which is consistent with the previous phylogenetic study (Fior et al., 2013). As an early‐acting reproductive barrier, ecogeographic isolation has long been viewed as the most important reproductive barrier in plants (Stebbins, 1950). Although geographic isolation might be limited during the speciation process, some degree of ecological isolation is a common element (Baack et al., 2015). Since ecogeographic isolation is essential between most species pairs, it is safe to presume that it is the first step toward eliminating the gene flow between our studied species by creating an ecological barrier. This helps in accumulating genetic differences and sharpening boundaries between them.

### 4.3 Climate change and ecogeographic isolation in future

Given that extrinsic barriers can break down under environmental perturbation, it is valuable to predict how ecogeographic isolation will be altered by climate changes. Currently, many land species are shifting poleward as well as to higher altitudes (Chen et al., 2011; Parmesan & Yohe, 2003). As species track suitable habitat, contact zones will emerge at the range margins when the leading edge of an advancing southern (or lower latitude) taxa advance faster than the lagging edge of a retreating northern (or higher latitude) sister taxa. This increases the risk of hybridization as it can cause species to merge, posing a serious threat to species diversity (Chunco, 2014). Therefore, understanding how climate change affects reproductive barriers is critical in predicting hybridization responses to climate change.

Montane species are expected to be sensitive to climate warming and undergo upslope shifts because climate changes appear to be pronounced at high elevations (Bradley et al., 2006). Our finding showed that temperature plays a crucial role in determining the distributions of *Aquilegia* species, making them vulnerable to climate changes. We used ENMs to predict *Aquilegia* species' distributions would change in response to climate changes. Our study revealed that, under different climate change scenarios, most of the studied *Aquilegia* plants would expand their range sizes at least once from the current to 2050 with climate warming. This finding is consistent with a recent study in the mountains of southwest China (Liang et al., 2018), which indicated that most species would expand their range sizes as they shift upslope in response to climate warming (Table 1). This suggested that more surface area became available due to upslope shifting (Elsen & Tingley, 2015), probably due to the complex topography of the mountains. Another modeling study on *Polylepis* species (Rosaceae) in the Andes Mountains similarly suggested that increased range sizes are likely to occur as a result of upward movement due to climate warming (Zutta & Rundel, 2017). As most species expand in range size, our results showed that climate changes are likely to increase the overall ecogeographic isolation between most species pairs in the future, with the exception of *A. kansuensis* and *A. ecalacarata*, which remain distributed in sympatry. We infer that other reproductive barriers must prohibit gene flow between them and call for further investigations to reveal these reproductive barriers. In a word, the findings discredit the possibility of climate‐mediated hybridization arising from solid ecogeographic barriers.

## 5 CONCLUSIONS

*Aquilegia* species usually form notable hybrid zones when they overlap or adjoin one another (Grant, 1952; Hodges & Arnold, 1994; Li et al., 2014). Our present work revealed that ecogeographic isolation plays a vital role in the diversification of *Aquilegia* species in the Mountains of northern and southwestern China, where many recent speciation events have been discovered (Wan et al., 2021). Under different climate change scenarios in the future, ecogeographic isolation would reduce gene flow because climate change increases ecogeographic isolation between closely related *Aquilegia* species. However, the overlapping between *A. kansuensis* and *A. ecalacarata* in three future climate scenarios is quite considerable, suggesting further study on other reproductive barriers between them is needed. The present results highlight that quantifying ecogeographic isolation is essential to understand the factors driving speciation and to help foresee potential hybridization in the future. Considering *Aquilegia* species are highly interfertile (reviewed in Kramer, 2009) and usually pollinated by generalist bees in Asia (e.g., Tang et al., 2007; Toji et al., 2022), weak ecogeographic isolation between *A. kansuensis* and *A. ecalacarata* leads to another question: how they remain strong enough reproductive isolated in sympatric populations? However, it was not considered in the present study, but merits further investigation.

## AUTHOR CONTRIBUTIONS

**Yulin Weng:** Data curation (equal); formal analysis (equal); writing – original draft (lead). **Huiqiong Li:** Data curation (equal); formal analysis (equal); investigation (equal). **Jiqin Yang:** Data curation (equal). **Zhi‐Qiang Zhang:** Funding acquisition (supporting); project administration (supporting); writing – review and editing (lead).

## Supporting information


Appendix S1.

## Data Availability

Datasets on occurrence records are available at: https://doi.org/10.5061/dryad.cnp5hqc95.
