# Supplementary material for: The past, present, and future of ecogeographic isolation between closely related Aquilegia plants
Source: Ecol Evol. 2023 May 25;13(5):e10098. doi: 10.1002/ece3.10098 (PMC10212700; doi:10.1002/ece3.10098)
Supplement: Supplementary file 1 — Appendix S1. [file ECE3-13-e10098-s001.docx]

**Table S1.** Environmental variables used to predict distribution of *A. ecalcarata*, *A. kansuensis*, *A. rockii* and *A. yabeana* in this study.

| Types of variables | Abbreviation | Environmental variables |
| --- | --- | --- |
|  | bio1 | Annual Mean Temperature |
|  | bio3 | Isothermality |
| Bioclimatic variables | bio7 | Temperature Annual Range |
|  | bio12 | Annual Precipitation |
|  | bio15 | Precipitation Seasonality |
| Radiation variables | srad12 | Solar radiation of December |

**Table S2.** Contributions of six environmental factors to principal components for four species. (The higher the absolute value, the greater the contribution)

|  | PC1 | PC2 | PC3 | PC4 |
| --- | --- | --- | --- | --- |
| Annual Mean Temperature | -0.4126 | -0.2224 | 0.8653 | -0.1099 |
| Isothermality | 0.2826 | 0.5384 | 0.1439 | -0.6299 |
| Temperature Annual Range | 0.3247 | -0.5614 | 0.0139 | 0.1137 |
| Annual Precipitation | -0.5523 | 0.1113 | -0.1660 | -0.2656 |
| Precipitation Seasonality | 0.4859 | -0.3187 | 0.1801 | -0.4631 |
| Solar radiation of December | 0.3215 | 0.4812 | 0.4128 | 0.5414 |


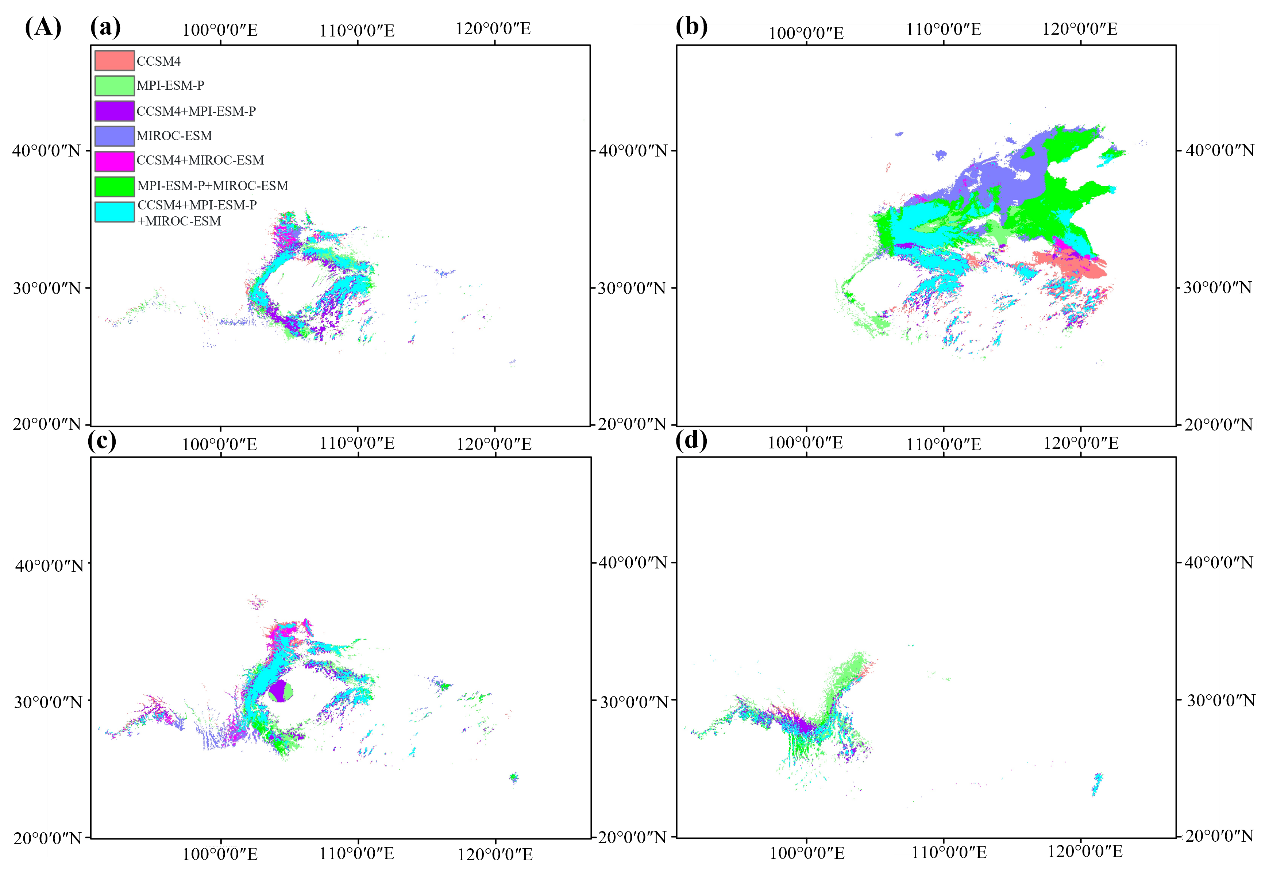


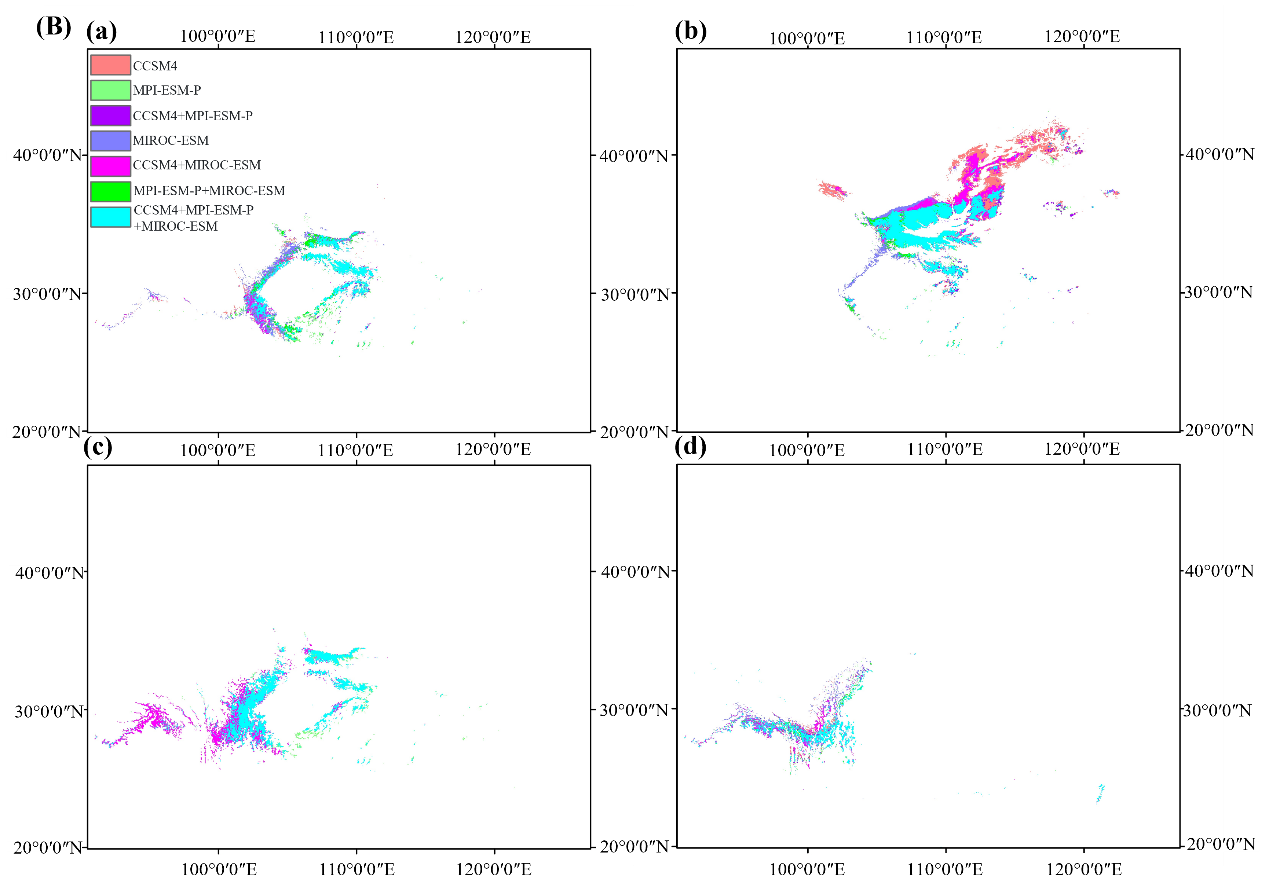


**Fig. S1.** The potential habitat of *A. ecalcarata*, *A. kansuensis*, *A. rockii* and *A. yabeana* predicted by species distribution models (SDMs) under three general circulation models (GCMs): CCSM4, MIROC-ESM, and MPI-ESM-P. (A) represent potential habitat of each species at LGM, (B) represent potential habitat of each species at MID; (a) represent species of *A. kansuensis,* (b) represent species of *A. yabeana,* (c) represent species of *A. ecalcarata*, (c) represent species of *A. rockii.*


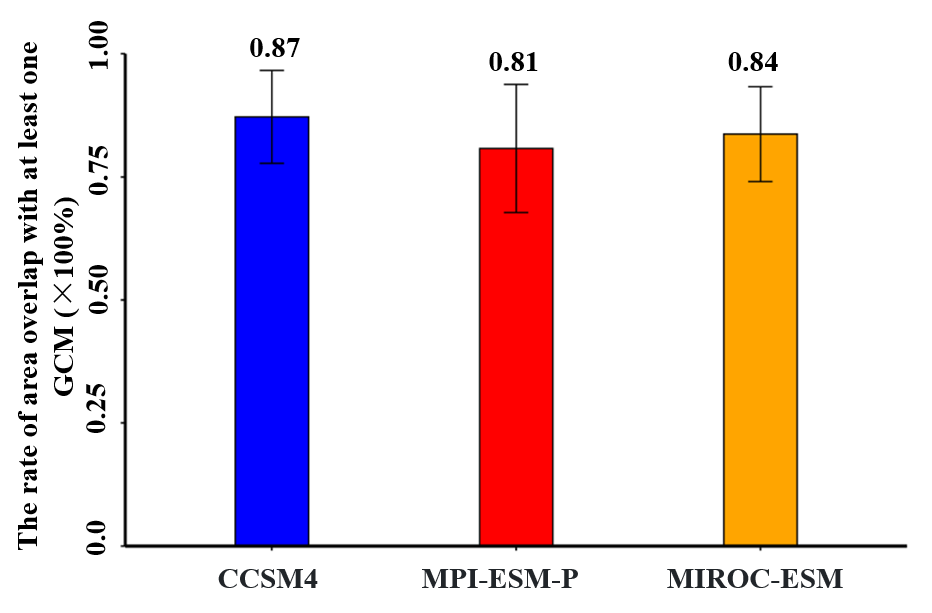


**Fig. S2.** The rate of area overlaps with at least one GCM in three GCMs.


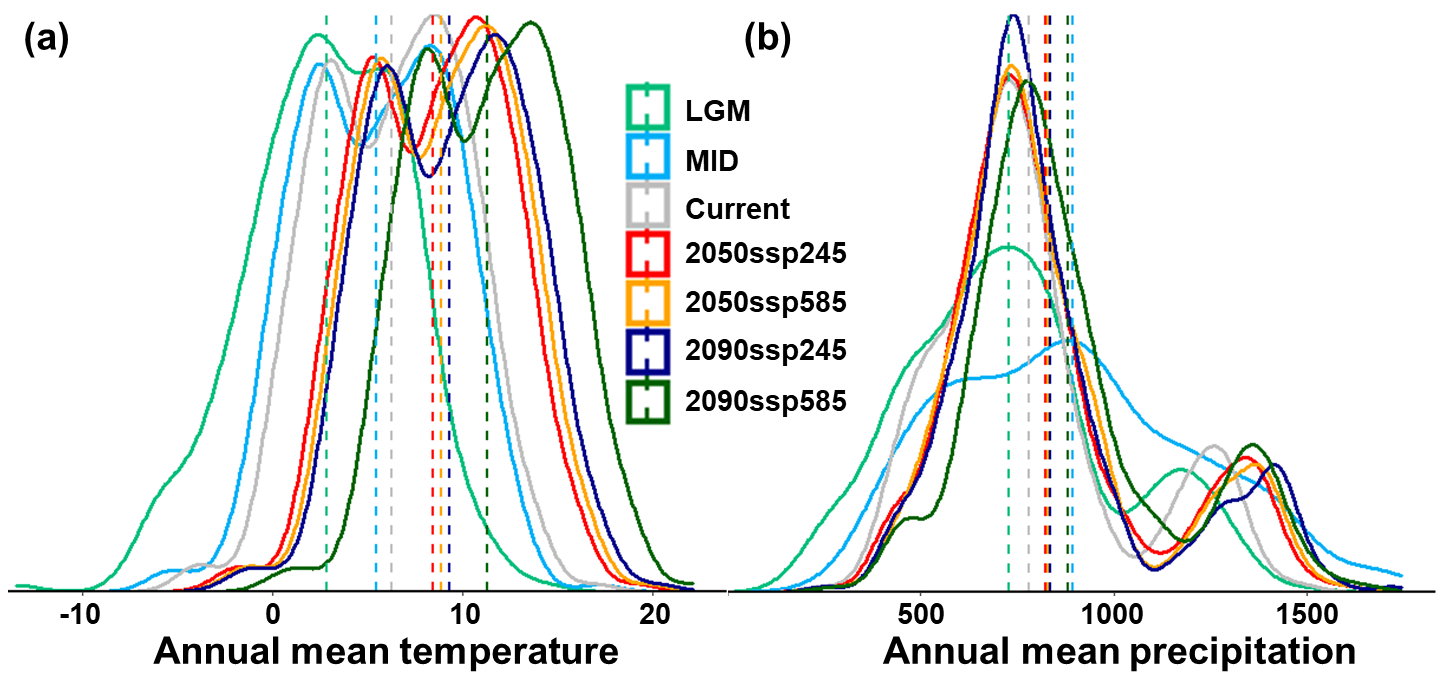


**Fig. S3.** Exemplar frequency curve of (a) annual mean temperature and (b) annual mean precipitation. The dotted line represents the average value and height of each curve corresponds to the proportion of the area in China with the corresponding temperature or precipitation level at past of LGM (green), MID (blue) and current (gray), future of 2050ssp245 (red), 2050ssp585 (orange), 2090ssp245 (dark-blue) and 2090ssp585 (dark-green).

*
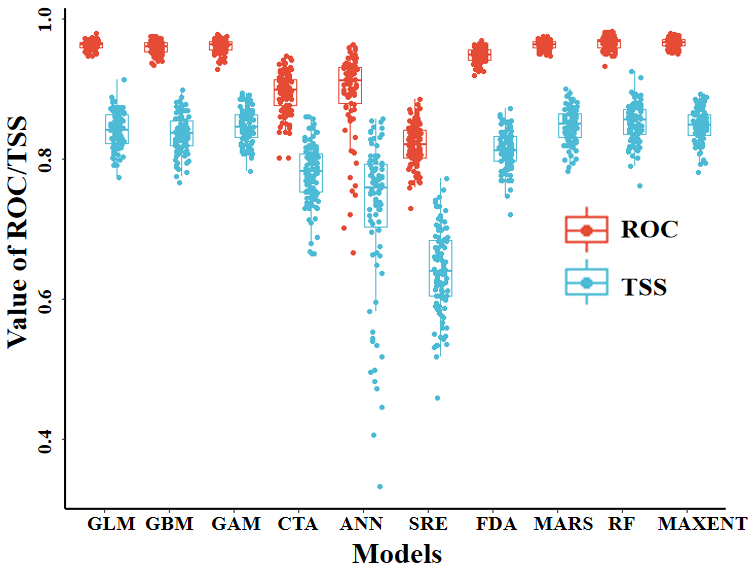
*

**Fig. S4.** Selection of species distribution models for *A. ecalcarata*, *A. kansuensis*, *A. rockii* and *A. yabeana* based on 7 environmental variables according to value of ROC (red) and TSS (blue). (Each model run 100 repeated).


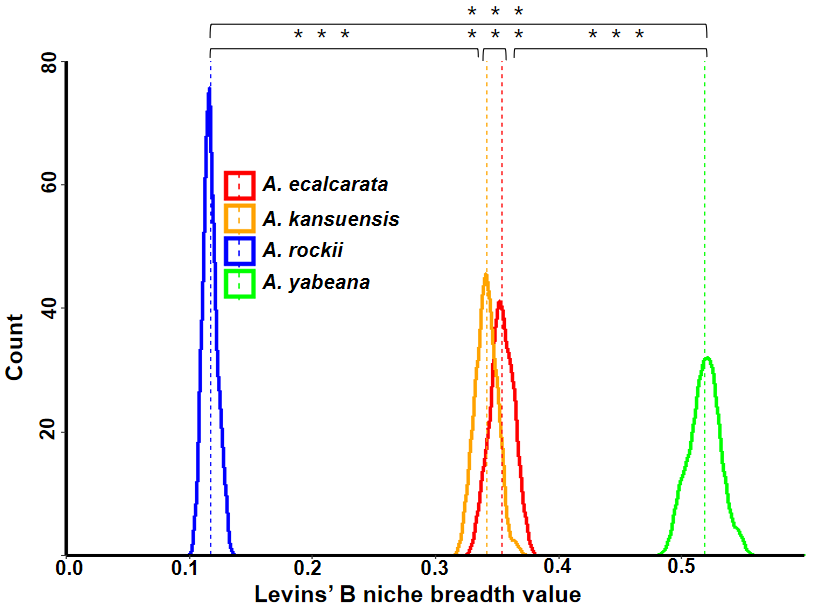


**Fig. S5.** Distribution of Levins’ *B* (niche breadth) values for sister species of *A. ecalcarata*, *A. kansuensis*, *A. rockii* and *A. yabeana*. Dashed lines indicate mean values, with P-values generated from two-tailed test nonparametric Mann-Whitney U test(＊＊＊;P<0.001), red curve represents *A. ecalcarata*, orange curve represents *A. kansuensis*, blue curve represents *A. rockii* and green curve represents *A. yabeana.* Each species has significantly difference niche breadth in this clade(P<0.001).


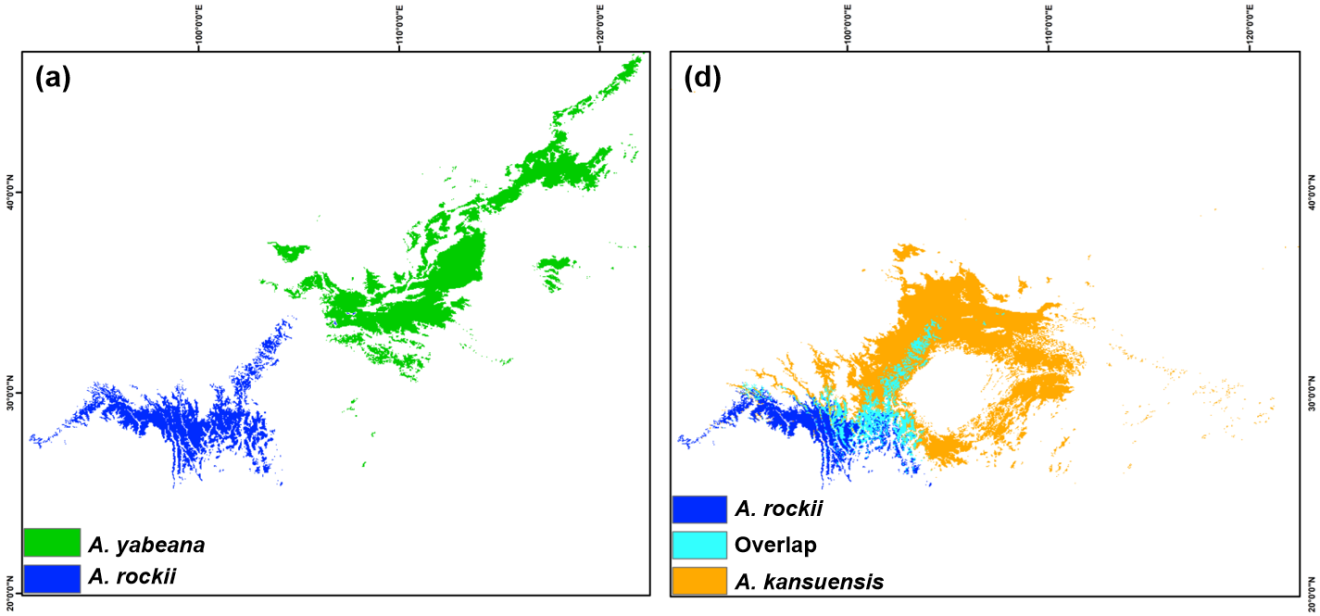


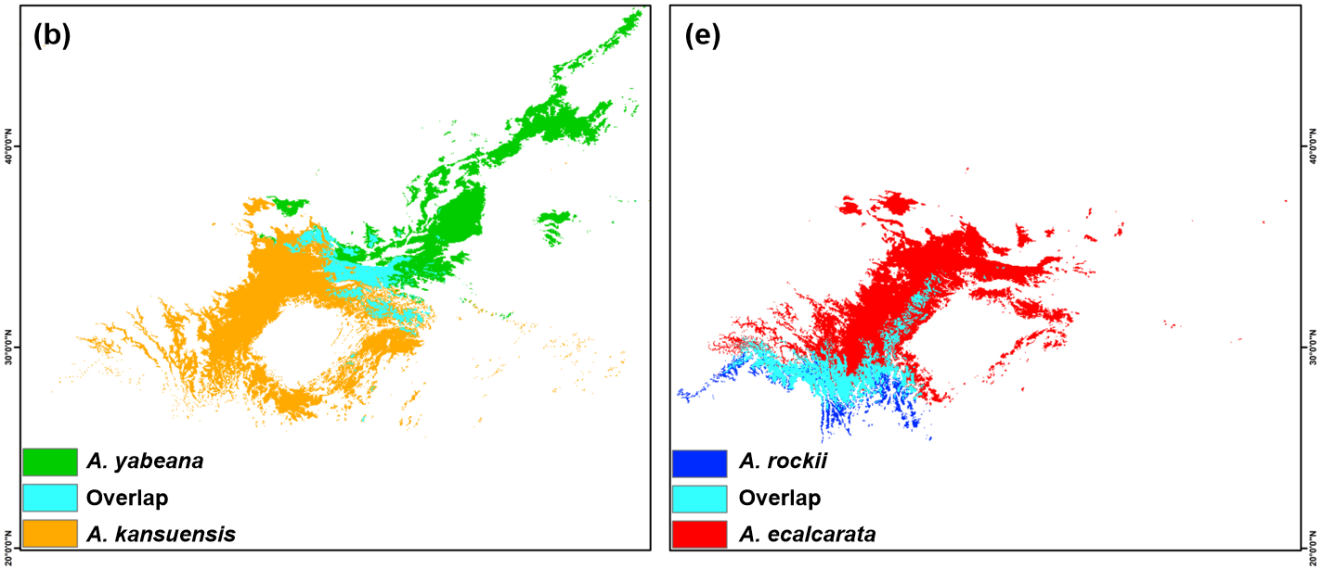


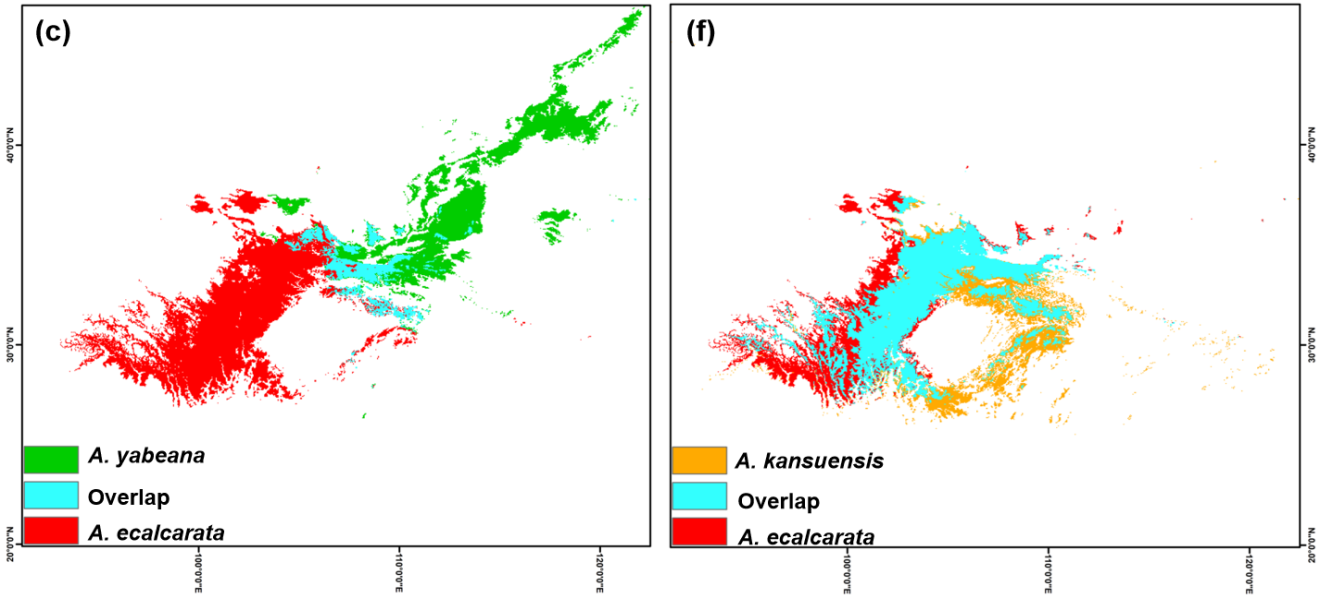


**Fig. S6.** The potential habitat and overlapping habitat of *A. ecalcarata*, *A. kansuensis*, *A. rockii* and *A. yabeana*. In (a) green represents the potential habitat of *A. yabeana*, blue represents the potential habitat of *A. rockii*, lightblue represents the overlapping habitat of two species; In (b) green represents the potential habitat of *A. yabeana*, orange represents the potential habitat of *A. kansuensis*, lightblue represents the overlapping habitat of two species; In (c) green represents the potential habitat of *A. yabeana*, red represents the potential habitat of *A. ecalcarata*, lightblue represents the overlapping habitat of two species; In (d) blue represents the potential habitat of *A. rockii*, orange represents the potential habitat of *A. kansuensis*, lightblue represents the overlapping habitat of two species; In (e) blue represents the potential habitat of *A. rockii*, red represents the potential habitat of *A. ecalcarata*, lightblue represents the overlapping habitat of two species; In (f) orange represents the potential habitat of *A. kansuensis*, red represents the potential habitat of *A. ecalcarata*, lightblue represents the overlapping habitat of two species.


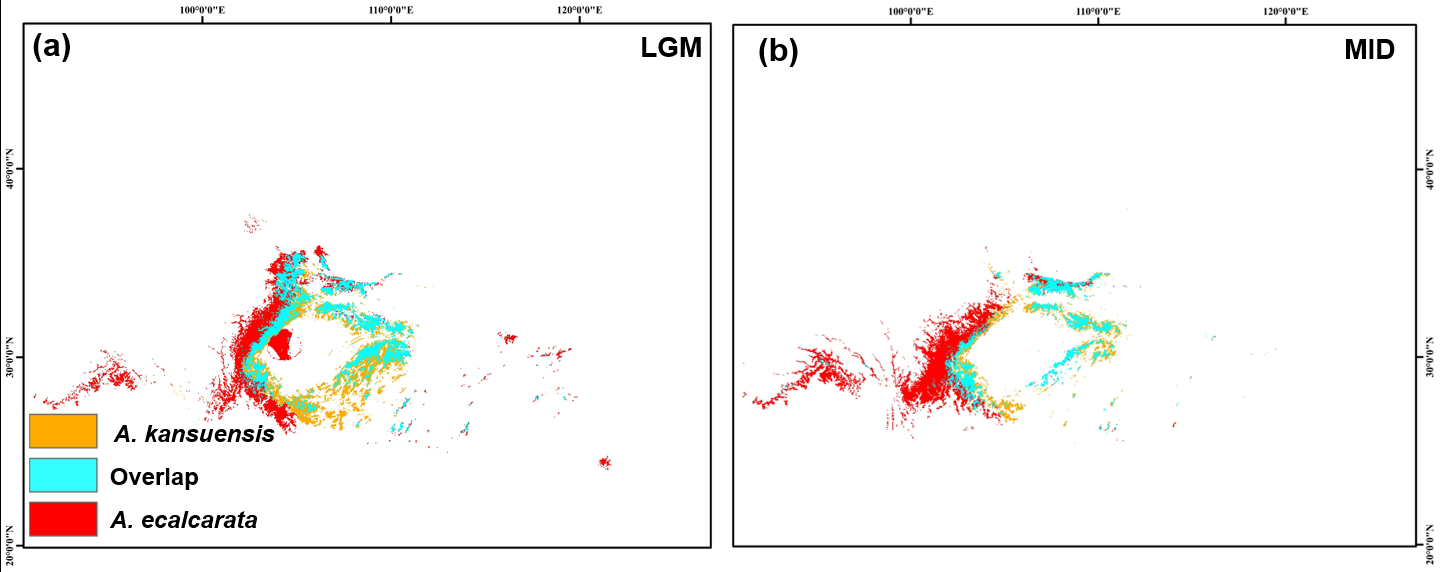


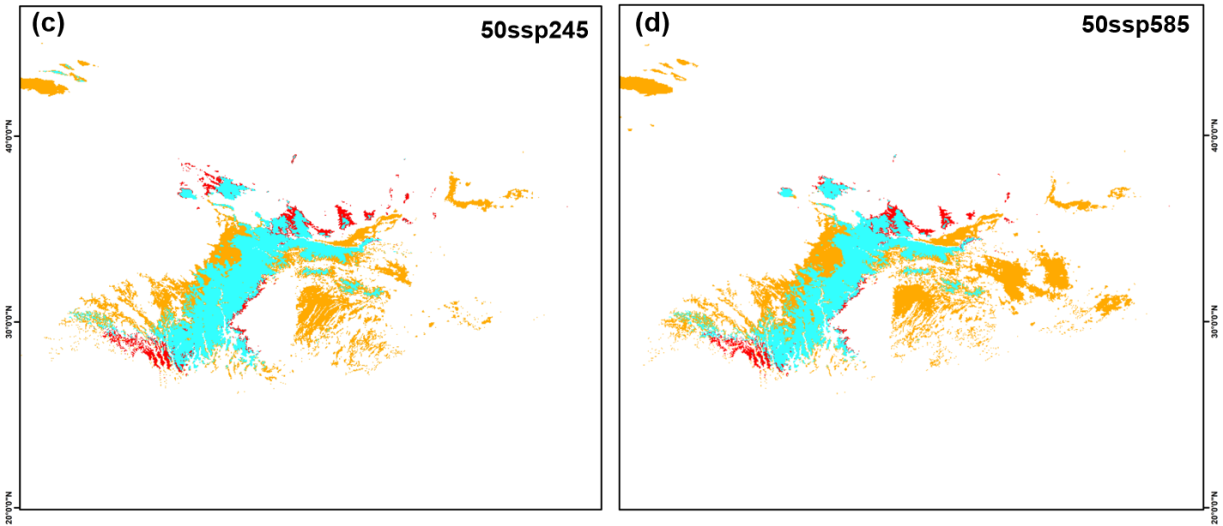


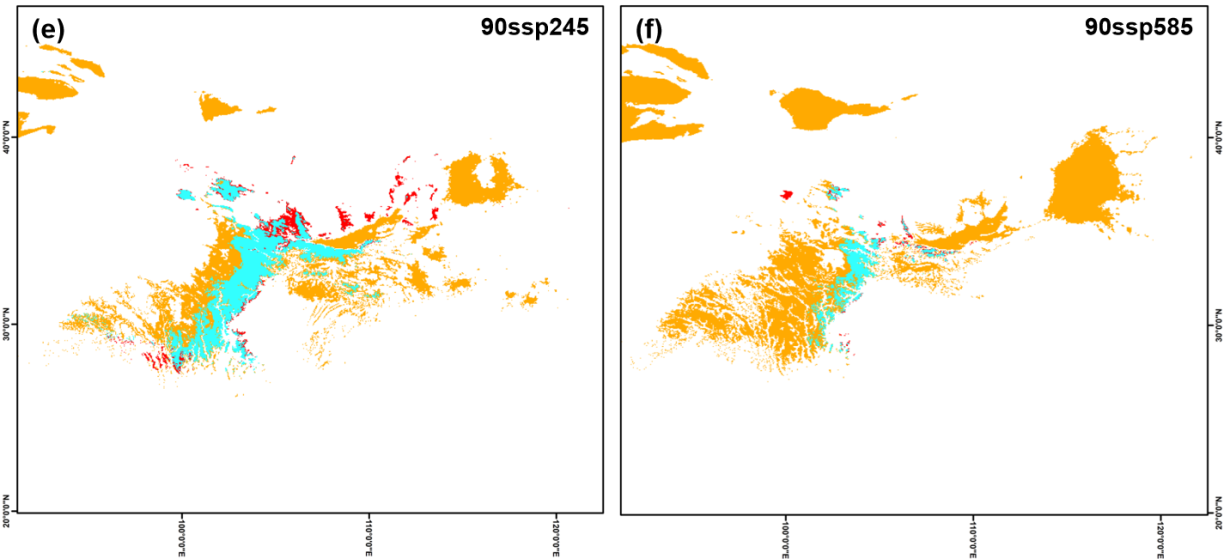


**Fig. S7.** The potential habitat and overlapping habitat of *A. ecalcarata* and *A. kansuensis* past, present, and all climate change scenarios in future. (a) Last Glacial maximum (LGM), (b) Mid Holocene (MID), (c) the 50ssp245, (d) 50ssp585, (e) 90ssp245, (f) 90ssp585.


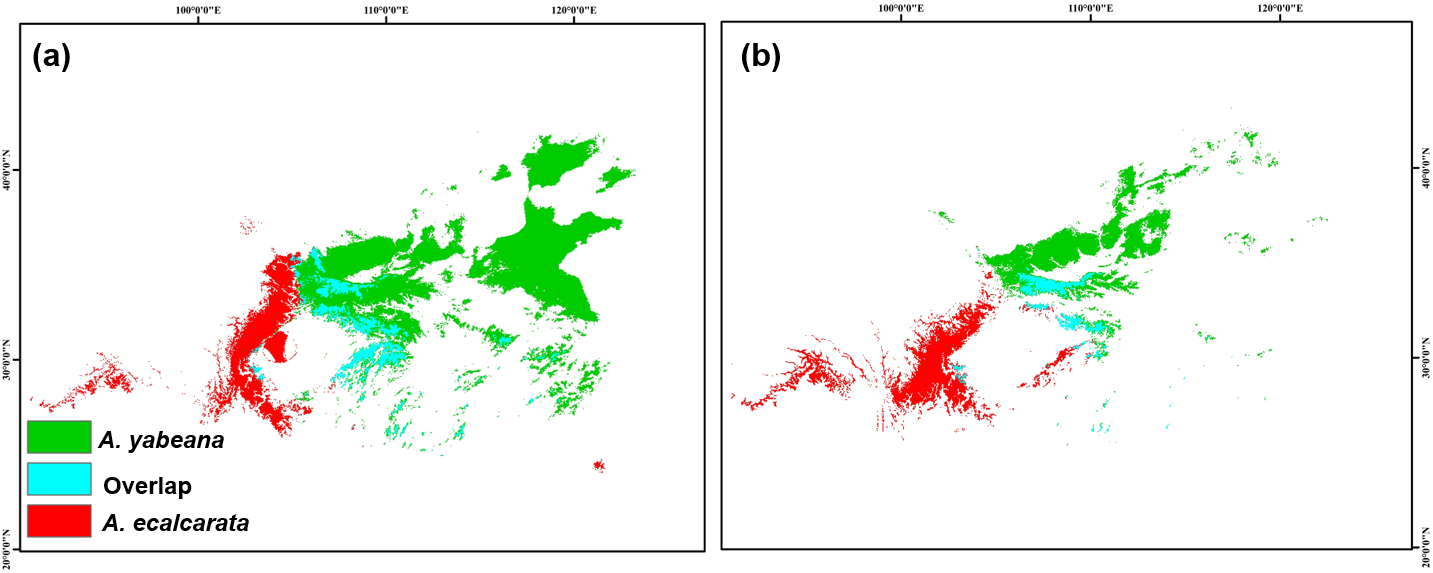


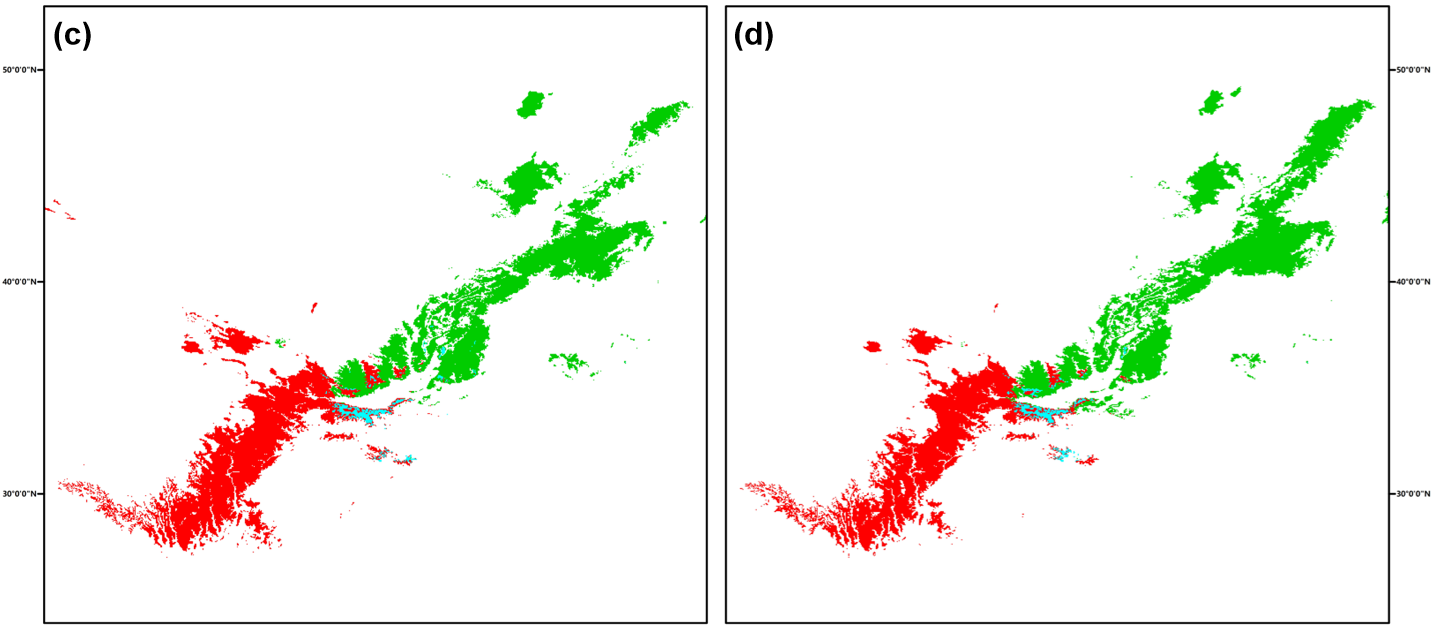


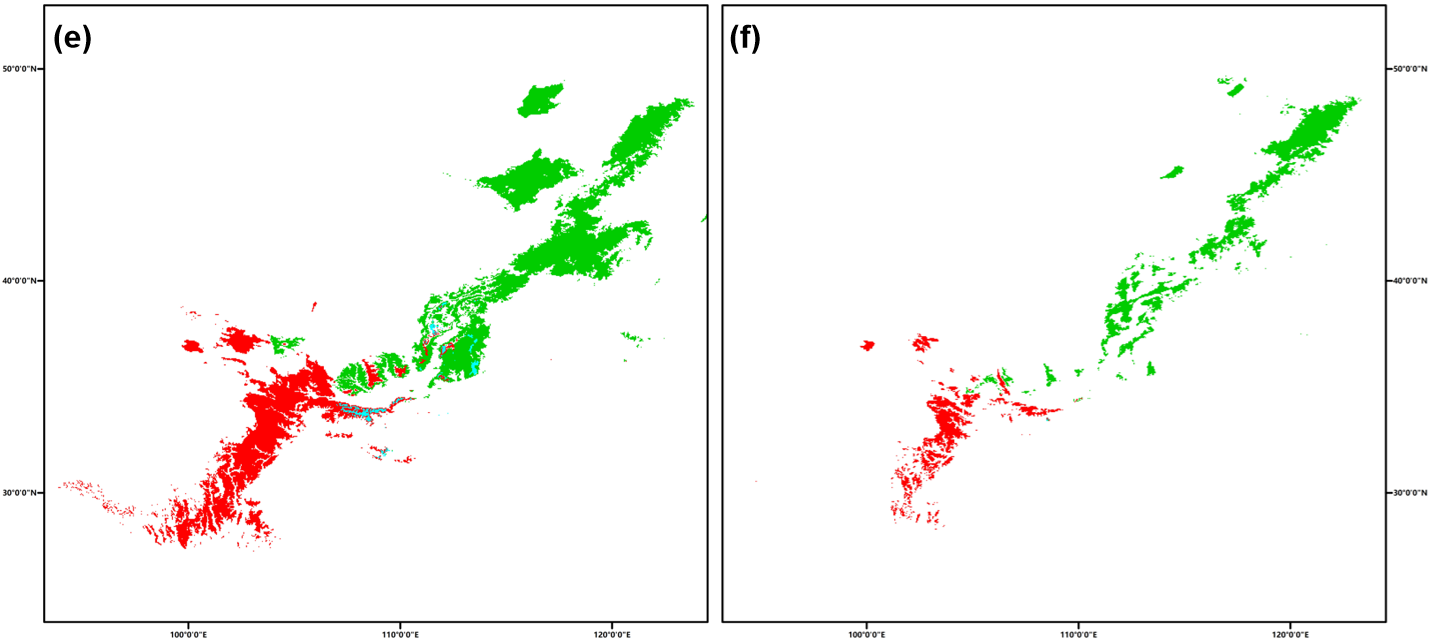


**Fig. S8.** The potential habitat and overlapping habitat of *A. ecalcarata* and *A. yabeana* for past, present, and all climate change scenarios in future. (a) Last Glacial maximum (LGM), (b) Mid Holocene (MID), (c) the 50ssp245, (d) 50ssp585, (e) 90ssp245, (f) 90ssp585.


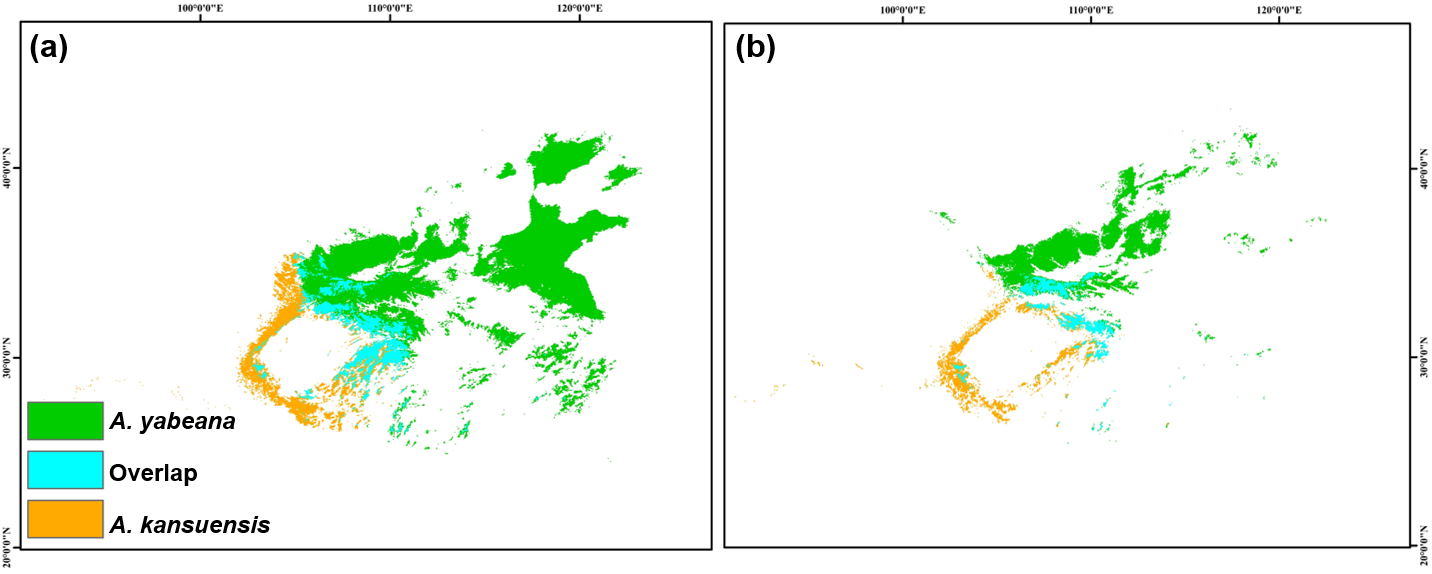


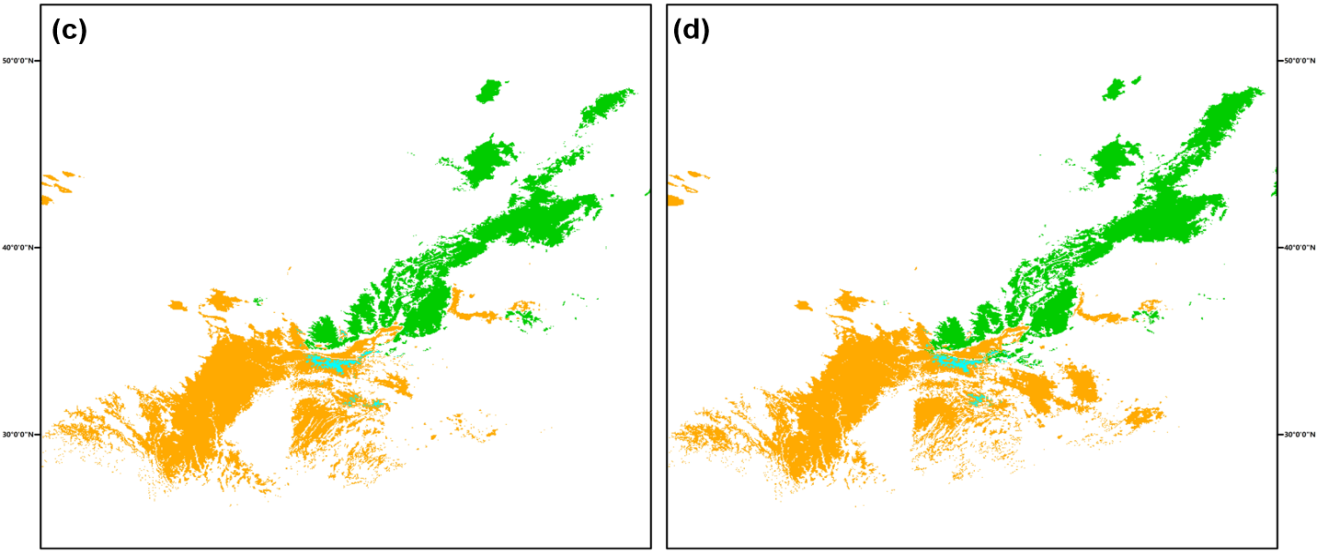


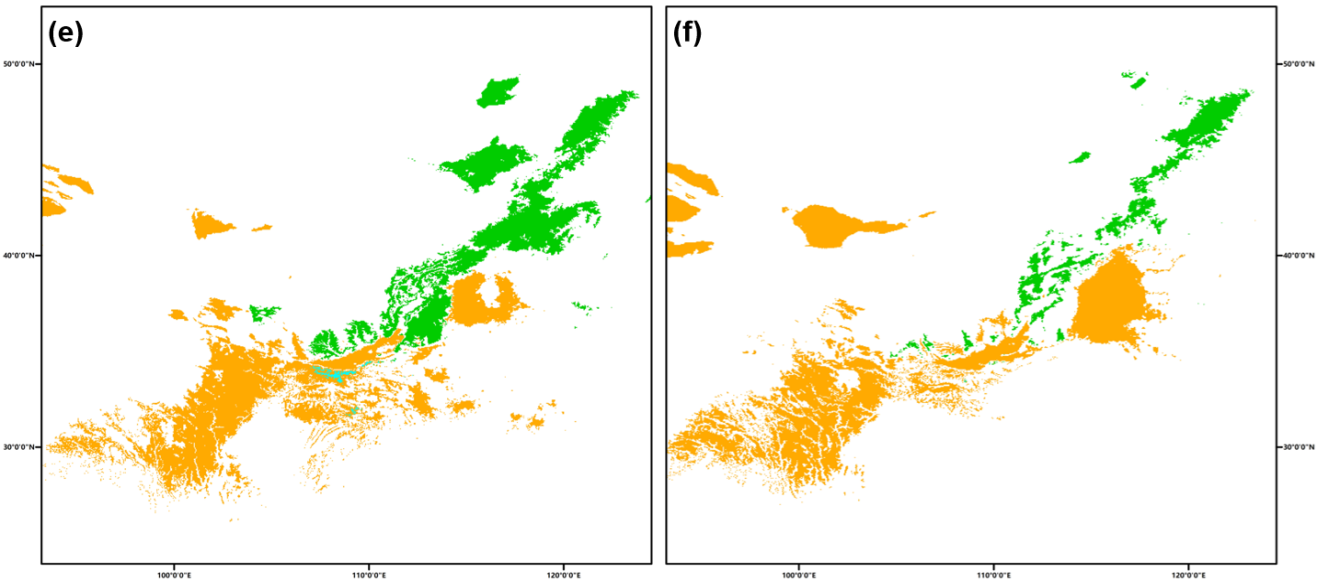


**Fig. S9.** The potential habitat and overlapping habitat of *A. kansuensis* and *A. yabeana* for past, present, and all climate change scenarios in future. (a) Last Glacial maximum (LGM), (b) Mid Holocene (MID), (c) the 50ssp245, (d) 50ssp585, (e) 90ssp245, (f) 90ssp585.

(For *A. rockii*, there were nearly no overlap with other species for past and all climate change scenarios in future, so figure about *A. rockii* overlap with other species were not showed)
